# Identifying Early-Stage Changes in Volatile Organic Compounds of *Ceratocystis fimbriata* Ellis & Halsted-Infected Sweet Potatoes (*Ipomoea batatas* L. Lam) Using Headspace Gas Chromatography-Ion Mobility Spectrometry

**DOI:** 10.3390/foods12112224

**Published:** 2023-05-31

**Authors:** Jiyu Cheng, Jiawen Wu, Zhihao Liu, Xiaoqiong Zhang, Xinghua Lu, Liqing Yin, Guoquan Lu, Linjiang Pang

**Affiliations:** 1 College of Food and Health, Zhejiang A&F University, Hangzhou 311300, China; cjy@zafu.edu.cn (J.C.); wjw_1006@163.com (J.W.); 2020601051011@stu.zafu.edu.cn (Z.L.); xqzhang@stu.zafu.edu.cn (X.Z.); xhlu@zafu.edu.cn (X.L.); 20220027@zafu.edu.cn (L.Y.); 2 Institute of Root & Tuber Crops, Zhejiang A&F University, Hangzhou 311300, China; lugq10@zju.edu.cn

**Keywords:** sweet potato, *C. fimbriata*, infect, HS-GC-IMS, VOCs, discrimination

## Abstract

*Ceratocystis fimbriata* Ellis & Halsted is the pathogen causing black rot in sweet potatoes that can lead to flavor change and toxin release. This study detected the volatile organic compounds (VOCs) of *C. fimbriata*-infected sweet potatoes in the early stages using headspace gas chromatography-ion mobility spectrometry (HS-GC-IMS). A total of 55 VOCs were identified, including aldehydes, alcohols, esters, ketones, and others. The content of aldehydes and ketones showed a decreasing trend, while alcohols and esters showed an increasing trend. An increase in infection time elevated the content of malondialdehyde (MDA) and pyruvate, while the starch content decreased, the content of soluble protein initially increased, then decreased, and the activities of lipoxygenase (LOX), pyruvate decarboxylase (PDC), alcohol dehydrogenase (ADH), and phenylalanine ammonia-lyase (PAL) increased. The changes in VOCs were closely related to the content of MDA, starch, pyruvate, and the activities of LOX, PDC, ADH, and PAL. Sweet potatoes showed a good discrimination effect by principal component analysis (PCA) and orthogonal partial least squares-discriminant analysis (OPLS-DA) from 0 to 72 h. Twenty-five differential VOCs could be used as early-stage characteristic compounds of *C. fimbriata*-infected sweet potatoes for early disease monitoring.

## 1. Introduction

Sweet potato (*Ipomoea batatas* L. Lam) is the sixth largest production crop in the world with a high yield and strong adaptability. It is widely planted in tropical and subtropical areas. The world’s sweet potato planting area was 7.41 million hectares in 2021 according to FAO statistics, with a total output of approximately 89 million tons. China’s planting area was 2.2 million hectares, with a total output of approximately 48 million tons. Sweet potato is regarded as a balanced and comprehensive healthy food since it is rich in starch, dietary fiber, vitamins, minerals (magnesium, iron, copper, manganese, calcium, potassium), and bioactive substances (phenolic acids, flavonoids, anthocyanins) [1].

Sweet potatoes have a high moisture content and fragile epidermis; therefore, they are easily infected by pathogens during postharvest storage and transportation that leads to decay and deterioration. Black rot is one of the main sweet potato diseases with approximately 10–20% of sweet potatoes lost to this disease [2]. This causes a great economic loss to the sweet potato industry. *Ceratocystis fimbriata* Ellis & Halsted (*C. fimbriata*) is the pathogen causing black rot through the invasion of sweet potato skin wounds. It produces a large amount of ipomeamarone toxin within 2 to 3 days after infection [3]. This is a potential hazard to human and livestock health. The accelerated respiratory rate of agricultural products infected by pathogens promotes the decomposition of various substrates and the synthesis of metabolites. This results in significant changes in the type and content of released volatile organic compounds (VOCs) that could be used as signal molecules to reflect the physiological status of agricultural products [4]. For example, the stem-end rot of citrus caused by *Lasiodiplodia theobromae* leads to changes in (+)-dihydrocarvone, Z-carveol, cis-beta-terpineol, D-carvone, and 3-methyl-2-buten-1-ol [5]. The green mold of mushrooms caused by *Trichoderma aggregativum* leads to changes in thujopsene, β-cadinene, patchulane, isocaryophyllene, and *β*-guaiene [6]. The soft rot of onions caused by *Erwinia carotovora* leads to changes in 1,2,4-trithiolane, acetic acid, hydrazide, eicosanoic acid, and ethyl ester [7]. Detecting the changes in VOCs after agricultural products are infected by pathogens is of great significance for the diagnosis and recognition of diseases.

Currently, headspace solid-phase microextraction gas chromatography-mass spectrometry (HS-SPME-GC-MS) is the most widely used technology for VOC detection. This includes measuring meat product flavor [8], tea grade identification [9], detection of pesticide residues in fruits [10], and detection of veterinary drug residues in fish [11]. However, complicated pre-treatments, long detection time, and the vacuum required for MS limits the portability of GC-MS. Headspace gas chromatography-ion mobility spectrometry (HS-GC-IMS) detects compounds colliding with the reverse-drift gas under the action of an electric field to produce ion groups. The compounds are identified according to the retention time and drift time [12]. This technique has the advantages of fast detection, high sensitivity, atmospheric pressure detection, and can provide visual images [13]. It was gradually used in the classification of oils, freshness assessment of fish, monitoring of wine processing, and identification of meat adulteration [14]. However, it has not been used to investigate VOC changes in the early stages of sweet potato infection by *C. fimbriata*.

This study assayed physiological indicators related to VOC synthesis to clarify their effect on VOCs and used HS-GC-IMS to determine the changes in VOCs from *C. fimbriata*-infected sweet potatoes in the early stages (0 to 72 h). Principal component analysis (PCA) and orthogonal partial least squares-discriminant analysis (OPLS-DA) models were used to discriminate sweet potatoes at different infection times and select differential VOCs. The results provided a new detection method for early disease monitoring and warning in sweet potatoes and laid a theoretical foundation for the in-depth study of the metabolic mechanism in sweet potato VOCs.

## 2. Materials and Methods

### 2.1. Sample Preparation

Sweet potatoes of the variety “Xinxiang” were provided by the sweet potato planting base of the crop research institute at Zhejiang A&F University in Hangzhou, China, and sampled in autumn. *C. fimbriata* pathogen was provided by the Xuzhou Academy of Agricultural Sciences in Xuzhou, China. Spore suspensions were prepared according to a previous method [15]. The spores were cultured on potato dextrose agar medium at 28 °C and 85% relative humidity for 7 to 10 days, rinsed with sterile saline solution, and the concentration was adjusted to 1 × 10^6^ spores/mL.

Fresh sweet potatoes of the same color and size without disease were selected for artificial inoculation. The sweet potatoes were washed with tap water, soaked in 1% *v*/*v* sodium hypochlorite solution for 2 min, rinsed with purified water, and wiped with 75% ethanol solution to disinfect the surface. Six holes were evenly drilled in the equator of sweet potatoes with a diameter of 2 mm and a depth of 5 mm. Each hole was inoculated with 10 µL of spore suspension and incubated for 5 min until complete absorption was observed. Sweet potatoes were placed in a constant temperature and humidity incubator at 28 °C and 80% relative humidity, and representative samples were taken every 8 h from 0 to 72 h. The epidermis and internal tissues were collected 1 to 1.5 cm away from the inoculation site for analysis.

### 2.2. Malondialdehyde, Starch, Soluble Protein, and Pyruvate Content Assays

Malondialdehyde (MDA) content assay: One gram of freeze-dried sweet potato sample was accurately weighed and homogenized in 5.0 mL 100 g/L trichloroacetic acid (TCA) solution pre-cooled at 4 °C. The homogenate was centrifuged at 12,000× *g* for 20 min at 4 °C. The supernatant was collected as MDA extract, and the assay was performed according to the method of Plazas et al. [16]. Three biological replicates were performed at each time point. The MDA content was presented as nmol/g dry weight (DW).

Starch content assay: One gram of freeze-dried sweet potato sample was accurately weighed and homogenized in 10 mL 85% *v*/*v* ethanol. Fat and soluble sugar were washed with petroleum ether and 85% *v*/*v* ethanol, respectively. Six mL of 6 M hydrochloric acid solution was added to the residue, hydrolyzed in boiling water for 1 h, centrifuged at 8000× *g* for 10 min at 25 °C, and the pH of the supernatant was adjusted to 7.0. The starch content assay was performed according to the Chinese official analysis method (Chinese Official Document number: GB5009.9-2016) [17]. Three biological replicates were performed at each time point. The starch content was presented as % DW.

Soluble protein content assay: Two hundred milligrams of freeze-dried sweet potato sample was accurately weighed and homogenized in 5 mL distilled water. The homogenate was centrifuged at 12,000× *g* for 20 min at 4 °C. The supernatant was collected as soluble protein extract and the assay was performed according to the method of Chu et al. [18]. Three biological replicates were performed at each time point. The soluble protein content was presented as mg/g DW.

Pyruvate content assay: Five hundred milligrams of freeze-dried sweet potato sample was accurately weighed and homogenized in 5 mL 80 g/L TCA solution pre-cooled at 4 °C. The homogenate was centrifuged at 12,000× *g* for 20 min at 4 °C. The supernatant was collected as pyruvate extract, and the assay was performed according to the method of Bacon et al. [19]. Three biological replicates were performed at each time point. The pyruvate content was presented as mg/100 g DW.

### 2.3. Lipoxygenase, Pyruvate Decarboxylase, Alcohol Dehydrogenase, and Phenylalanine Ammonia Lyase Activity Assays

Lipoxygenase (LOX) activity assay: One gram of fresh sweet potato sample was accurately weighed and homogenized in 5 mL extraction solution at 4 °C containing 0.1 M phosphate buffer with 1% *v*/*v* Triton X-100 and 4% *w*/*v* polyvinylpolypyrrolidone (PVPP) at pH 6.8. The homogenate was centrifuged at 12,000× *g* for 20 min at 4 °C. The supernatant was collected as LOX extract and the assay was performed according to the method of Cai et al. [20]. The increase of 0.01 in absorbance at 234 nm per minute per gram fresh weight (FW) was expressed as one unit (U) of LOX activity. Three biological replicates were performed at each time point, and LOX activity was presented as U/g FW.

Pyruvate decarboxylase (PDC) activity assay: One gram of sweet potato sample was accurately weighed and homogenized in 5 mL extraction solution containing 0.1 M MES buffer with 2 mM dithiothreitol (DTT) and 4% PVPP at pH 6.5 and 4 °C. The homogenate was centrifuged at 12,000× *g* for 20 min at 4 °C. The supernatant was collected as PDC enzyme extract, and the assay was performed according to the method of Zuo et al. [21]. The decrease of 0.01 in absorbance at 340 nm per minute per gram fresh weight was expressed as one U of PDC enzyme activity. Three biological replicates were performed at each time point, and PDC enzyme activity was presented as U/g FW.

Alcohol dehydrogenase (ADH) activity assay: The preparation of enzyme extract was the same as that for PDC. The assay was performed according to the method of Zhao et al. [22]. The decrease of 0.01 in absorbance at 340 nm per minute per gram fresh weight was expressed as one U of ADH enzyme activity. Three biological replicates were performed at each time point, and ADH enzyme activity was presented as U/g FW.

Phenylalanine ammonia-lyase (PAL) activity assay: One gram of sweet potato sample was accurately weighed and homogenized in 5 mL extraction solution containing 0.1 M boric acid buffer with 2 mM ethylenediaminetetraacetic acid (EDTA), 4% polyvinyl pyrrolidone (PVP), and 5 mmol/L β-mercaptoethanol at pH 8.8 and 4 °C. The homogenate was centrifuged at 12,000× *g* for 20 min at 4 °C. The supernatant was collected as PAL extract, and the assay was performed according to the method of Astaneh et al. [23]. The increase of 0.01 in absorbance at 290 nm per hour per gram of fresh weight was expressed as one U of PAL activity. Three biological replicates were performed at each time point, and PAL activity was presented as U/g FW.

### 2.4. HS-GC-IMS Analysis

Volatile organic compounds from *C. fimbriata*-infected sweet potatoes were analyzed by HS-GC-IMS (FlavourSpec^®^, Gesellschaft fur Analytische Sensorsysteme mbH, Dortmund, Germany) equipped with a PAL, RSI automatic sampler (CTC Analytics AG Company, Zwingen, Switzerland). The detection method was a modified version of Yao et al. [24]. One gram of sweet potato sample was accurately weighed, transferred into a 20 mL headspace vial, sealed, and incubated under oscillating heating mode at 60 °C at 500 r/min for 20 min. The headspace gas was injected by an automatic sampler with an injection volume of 200 μL, and an injector temperature of 65 °C.

Volatile organic compounds were separated by gas chromatography using an FS-SE-54-CB-1 capillary column (15 m × 0.53 mm, 1 μm) and coupled to the IMS at 60 °C. Nitrogen (99.999% purity) was used as the carrier gas under the following program: the initial flow rate of 2 mL/min was maintained for 2 min, then increased to 10 mL/min in 8 min, and finally increased to 100 mL/min in 15 min, and maintained for 5 min. The total detection time was 30 min. The analytes were ionized in an IMS ionization chamber containing a tritium ionization source in positive ion mode. Nitrogen (99.999% purity) was set as the drift gas for the IMS at a flow rate of 150 mL/min, and the drift tube temperature was 45 °C. 

The retention index of VOCs was calculated by laboratory analytical viewer (LAV) software using C_4_-C_9_ n-ketones (Sinopharm Chemical Reagent Beijing Co., Ltd., Beijing, China) as external references. The VOCs were identified by comparing the retention time and drift time of standards in the National Institute of Standards and Technology (NIST) library and IMS database retrieval software. Two biological replicates were performed at each time point, and the results were presented as the mean ± standard deviation.

### 2.5. Statistical Analysis

Three-dimensional (3D) topographic plots and two-dimensional (2D) topographic plots were compared by means of the Reporter plug-in in the HS-GC-IMS. The Gallery Plot plug-in was used for fingerprint comparison of VOC content. Analysis of variance (ANOVA) was carried out to evaluate the differences between samples using IBM SPSS Statistics 22, and *p* < 0.05 indicated statistically significant differences using Duncan’s multiple range tests. Origin 2023 was used for plotting and correlation analysis. PCA analysis and OPLS-DA were used for discrimination using Simca 14.1 software.

## 3. Results and Discussion

### 3.1. The Effects of C. fimbriata-infected Sweet Potatoes on Physiological Indicators Related to VOCs

#### 3.1.1. Changes in MDA, Starch, Soluble Protein, and Pyruvate Content

**Figure 1:**
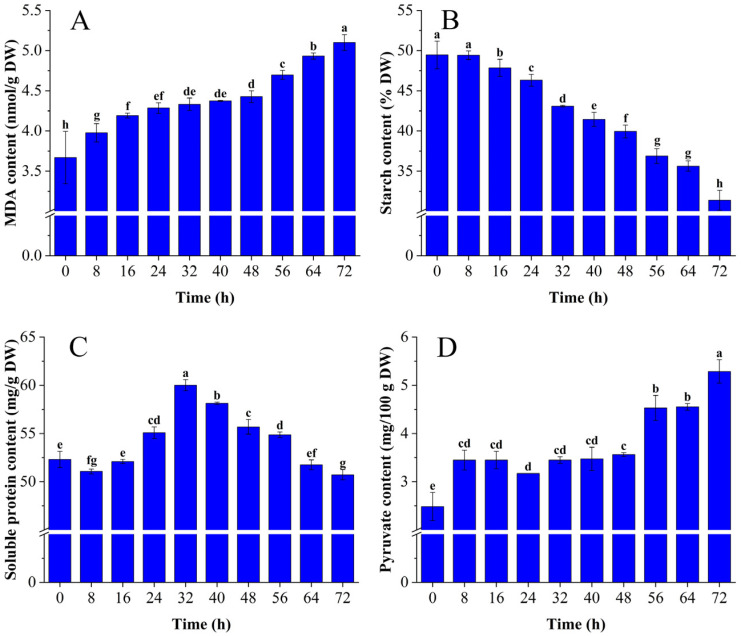
Changes of malondialdehyde (MDA) (**A**), starch (**B**), soluble protein (**C**), and pyruvate (**D**) content in *C. fimbriata*-infected sweet potatoes from 0 to 72 h. The bars represent the standard deviation of three independent measurements. Different lowercase letters represent significant differences according to Duncan’s multiple range test (*p* < 0.05).

The content of MDA, starch, soluble protein, and pyruvate significantly changed during *C. fimbriata* infection of sweet potato (*p* < 0.05). Malondialdehyde was the main product of membrane lipid peroxidation. The MDA content showed a gradually increasing trend from 0 to 72 h (Figure 1A) and increased by 39.05% in sweet potatoes infected for 72 h compared with fresh sweet potatoes (0 h). This was consistent with the changing trend of pepper infected by Phytophthora capsici [25]. The accumulation of MDA content reflected the increasing degree of oxidation of the membrane lipid and the gradual loss of biofilm integrity [26]. The increase in membrane permeability would lead to a change in fatty acid content that provides sufficient precursors for the synthesis of aldehydes, alcohols, and esters related to the LOX pathway. Starch is the main nutrient in sweet potatoes. The starch content showed a gradually decreasing trend from 0 to 72 h (Figure 1B) and decreased by 36.58% in sweet potatoes infected with *C. fimbriata* for 72 h compared with fresh sweet potatoes. This may be owing to the decomposition and consumption of starch by the growth and reproduction of *C. fimbriata*. This result was consistent with the changing trend of starch in maize infected by Fusarium verticillioides [27]. The glucose produced by starch decomposition could be degraded into pyruvate and acetyl-coA through glycolysis. This provides precursors for the synthesis of terpenoids in the methylerythritol 4-phosphate (MEP) pathway and the mevalonic acid (MVA) pathway [28]. Meanwhile, the soluble protein content initially increased, then decreased in sweet potatoes (Figure 1C). The soluble protein content increased by 14.72% in sweet potatoes infected by *C. fimbriata* for 32 h compared with fresh sweet potatoes. This may be owing to the stress response of sweet potatoes to *C. fimbriata*, which increased the activities of various defense enzymes resulting in increased protein content at the infected site. The soluble protein content decreased by 15.46% in sweet potatoes infected with *C. fimbriata* for 72 h compared with sweet potatoes infected for 32 h; this may be owing to the decomposition of soluble protein into amino acids. Some amino acids are important precursors for the synthesis of VOCs such as aldehydes, alcohols, acids, and esters in agricultural products [29]. Pyruvate is the glycolysis product from glucose. The pyruvate content showed a gradually increasing trend from 0 to 72 h and increased by 112.96% in sweet potatoes infected with *C. fimbriata* for 72 h compared with fresh sweet potatoes (Figure 1D). Pyruvate is in the metabolic pathway of ethanol fermentation and may be converted into acetaldehyde by PDC, and further metabolized into ethanol by ADH [30].

#### 3.1.2. Changes in LOX, PDC, ADH, and PAL Activity

**Figure 2:**
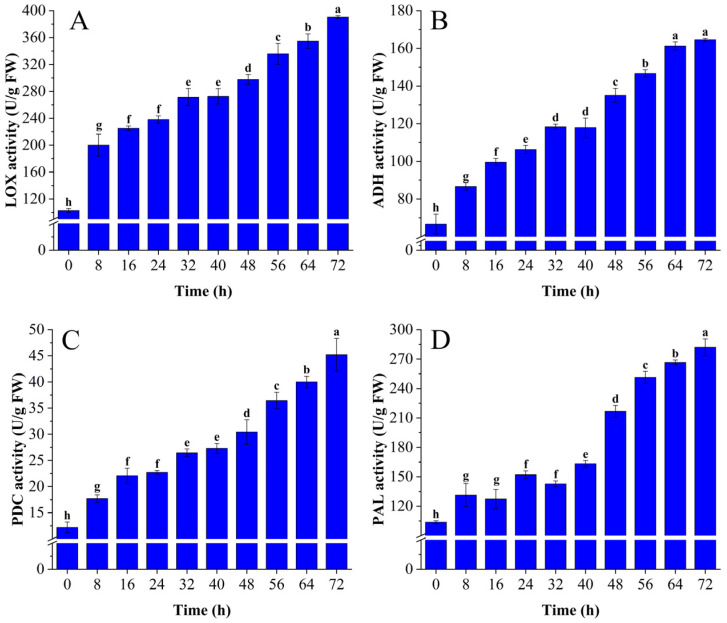
Changes of lipoxygenase (LOX) (**A**), pyruvate decarboxylase (PDC) (**B**), alcohol dehydrogenase (ADH) (**C**), and phenylalanine ammonia-lyase (PAL) (**D**), and activities in *C. fimbriata*-infected sweet potatoes from 0 to 72 h. Different lowercase letters represent significant differences according to Duncan’s multiple range test (*p* < 0.05).

The activities of LOX, PDC, ADH, and PAL significantly increased during *C. fimbriata* infection of sweet potato (*p* < 0.05). Lipoxygenase is the key enzyme catalyzing lipid peroxidation in cell membranes. Its activity showed a gradually increasing trend from 0 to 72 h (Figure 2A) and increased by 279.19% in sweet potatoes infected by *C. fimbriata* for 72 h compared with fresh sweet potatoes. This was consistent with the changing trend of bananas infected by *Fusarium oxysporum* [31]. The increase of LOX activity accelerates the conversion of unsaturated fatty acids into C_6_ and C_9_ aldehydes and alcohols and finally converts them into esters [32]. It also accelerates MDA synthesis. PDC is the key enzyme of the amino acid metabolism pathway of agricultural products. The PDC activity showed a gradually increasing trend from 0 to 72 h (Figure 2B) and increased by 270.94% in sweet potatoes infected for 72 h compared with fresh sweet potatoes. This was consistent with the changing trend of *Arabidopsis thaliana* infected by *Ralstonia solanacearum* [33]. The increase in PDC activity may accelerate the decarboxylation of α-ketoacid to aldehydes, and synthesize alcohols and esters through reduction and esterification [34]. Alcohol dehydrogenase is the key enzyme metabolizing short-chain alcohols in agricultural products. The ADH activity showed a gradually increasing trend from 0 to 72 h (Figure 2C) and increased by 146.88% in sweet potatoes infected with *C. fimbriata* for 72 h compared with fresh sweet potatoes. The increase in ADH activity may accelerate the conversion of aldehydes to alcohols [35]. Phenylalanine ammonia-lyase is the key enzyme in the phenylalanine metabolism pathway. The PAL activity showed a gradually increasing trend from 0 to 72 and increased by 172.03% in sweet potatoes infected with *C. fimbriata* for 72 h compared with fresh sweet potatoes (Figure 2D). This was consistent with the changing trend of PAL activity in citrus infected by citrus bent leaf viroid [36]. The increase in PAL activity could accelerate the non-oxidative deamination of phenylalanine to produce a variety of phenylethyl compounds, benzenoids, phenylpropanes, and phenylpropenes [37].

### 3.2. HS-GC-IMS Analysis of C. fimbriata-Infected Sweet Potatoes in the Early Stage

#### 3.2.1. Topographic Plots of *C. fimbriata*-Infected Sweet Potatoes

**Figure 3:**
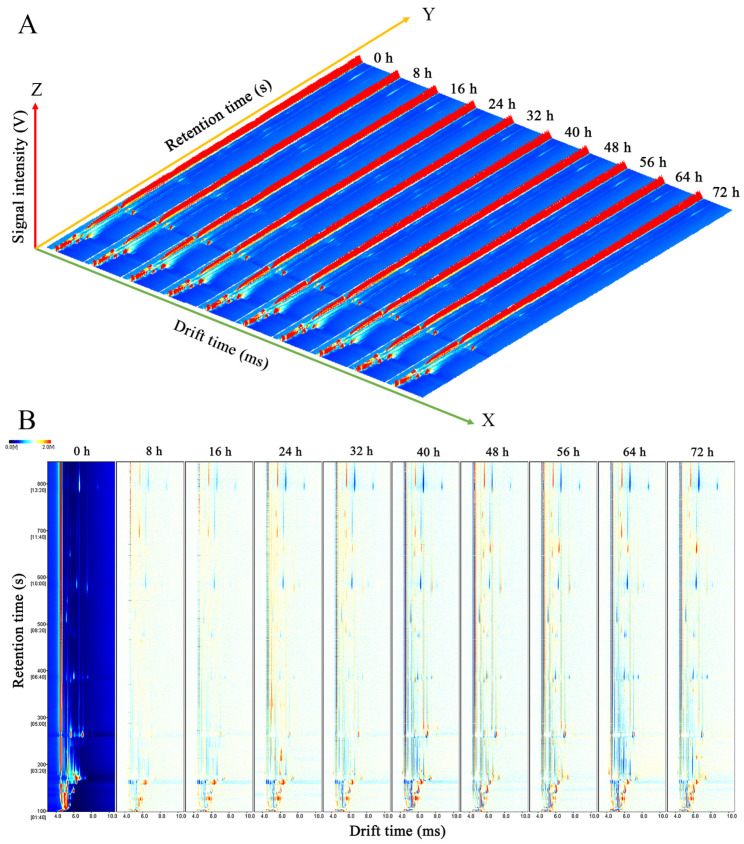
Three-dimensional topographic plots (**A**) and two-dimensional topographic plots (**B**) of VOCs in *C. fimbriata*-infected sweet potatoes from 0 to 72 h using headspace gas chromatography-ion mobility spectrometry (HS-GC-IMS).

The 3D topographic plots of VOCs in *C. fimbriata*-infected sweet potatoes at different times were shown in Figure 3A; the X axes, Y axes, and Z axes represented drift time (DT), retention time (RT), and ion peak intensity, respectively [38]. The 3D topographic plots from left to right represented sweet potatoes with different infection times from 0 to 72 h, respectively. The signal intensity of the 3D topographic plots of VOCs in *C. fimbriata*-infected sweet potatoes was different with increasing infection time, but the change was not obvious (Figure 3A). Therefore, the 3D plots were projected to corresponding 2D plots; the spectrogram of fresh sweet potatoes at 0 h was set as a reference and other samples were subtracted by the reference (Figure 3B). This was a more intuitive way to show the change in VOC content. The red and blue spots indicated that the concentration was higher and lower than the reference, respectively [39]. Most of the signals appeared between the drift time of 4.0 to 8.0 ms and the retention time of 100 to 800 s. The number of red and blue spots increased, and the color significantly deepened with the increase in infection time. This indicated that the VOC content changed at different infection times.

#### 3.2.2. Fingerprints of *C. fimbriata*-Infected Sweet Potatoes

**Figure 4:**
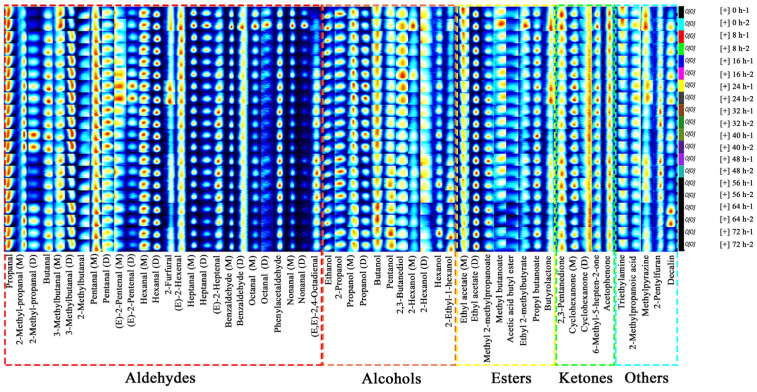
Fingerprints of HS-GC-IMS in *C. fimbriata*-infected sweet potatoes from 0 to 72 h.

The differences of all identified VOCs during the infection of sweet potatoes by *C. fimbriata* were analyzed by comparing the fingerprints (Figure 4). Each row represented the entire signal peak of each sweet potato sample, and each column represented the signal intensity of the same compound in different sweet potato samples. The darker red and blue colors represent higher and lower content, respectively [40]. Some compounds produced two signal peaks that were caused by the monomer (M) and dimer (D) forms [41]. The types of VOCs in sweet potatoes with different infection times were approximately the same, while the content was significantly different. Among them, the content of heptanal (M and D), octanal (M and D), nonanal (M and D), ethanol, ethyl acetate (M), 2,3-pentanedione, triethylamine, and methylpyrazine was relatively high in fresh sweet potatoes, and the content of 2-methyl-propanal (M and D) and (E,E)-2,4-octadienal was relatively low. The content of (E)-2-pentenal (D), 2-furfural, butyrolactone, 6-methyl-5-hepten-2-one, acetophenone, and 2-methylpropanoic acid was relatively high in sweet potatoes infected for 24 h, and the content of 2-hexanol (M) was relatively low. The content of (E,E)-2,4-octadienal, 2-propanol, 2-hexanol (D), methyl 2-methylpropanoate, and 2-pentylfuran were relatively high in sweet potatoes infected for 48 h, and the content of pentanal (M) and butanol was relatively low. The content of pentanol, hexanol, 2-ethyl-1-hexanol, and ethyl acetate (D) was relatively high in sweet potatoes infected for 72 h, and the content of (E)-2-pentenal (M and D), (E)-2-hexenal, heptanal (M and D), octanal (M and D), nonanal (M and D), 2,3-butanediol, acetic acid butyl ester, and 2-methylpropanoic acid was relatively low.

#### 3.2.3. Changes of VOC Content in *C. fimbriata*-Infected Sweet Potatoes

A total of 55 VOCs were identified in *C. fimbriata*-infected sweet potatoes from 0 to 72 h (Table 1). This includes 26 aldehydes, 11 alcohols, 8 esters, 5 ketones, and 5 others, with compounds containing monomers and dimers. 

The content of (E,E)-2,4-octadienal, 2-methyl-propanal (M and D), pentanol, 2-ethyl-1-hexanol, 3-methylbutanal (M and D), hexanol, propanol (M and D), and ethyl acetate (M and D) increased by 73.48%, 44.06%, 35.25%, 27.03%, 19.04%, 16.33%, 15.31%, and 10.87%, respectively in sweet potatoes infected for 72 h compared with fresh sweet potatoes. Among them, 2-methyl-propanal (M and D) and 3-methylbutanal (M and D) of branched-chain aldehydes were mainly derived from Strecker degradation of leucine and valine [42] by deamination and decarboxylation. Hexanol was mainly derived from the decomposition of unsaturated fatty acids such as linolenic acid and linoleic acid, and the increase of its content was affected by pathogen infection [43]. Ethyl acetate (M and D) had the highest content among esters. It is mainly derived from ethanol metabolism in sweet potatoes, which produced a pungent and “overripe” odor [44].

The content of nonanal (M and D), octanal (M and D), ethyl 2-methylbutyrate, 2-hexanol (M and D), heptanal (M and D), benzaldehyde (M and D), methyl butanoate, methylpyrazine, (E)-2-hexenal, triethylamine, 2,3-butanediol, 2-methylpropanoic acid, 2-pentylfuran, 2-furfural, butyrolactone, acetic acid butyl ester, ethanol, and (E)-2-pentenal (M and D) decreased by 53.67%, 48.91%, 35.91%, 34.01%, 33.41%, 26.06%, 20.93%, 20.10%, 18.25%, 17.19%, 17.06%, 14.61%, 13.67%, 13.25%, 12.45%, 11.72%, 11.20%, and 10.14%, respectively in sweet potatoes infected for 72 h compared to fresh sweet potatoes. Among them, nonanal (M and D), octanal (M and D), and (E)-2-hexenal of straight-chain aldehydes were oxidized derivatives of unsaturated fatty acids that were mainly derived from fatty acid metabolism of agricultural products [45]. In particular, (E)-2-hexenal increased the mRNA levels of genes encoding ethylene receptors, LOX, and PAL [46] to enhance the resistance of sweet potatoes. Methyl butanoate, butyrolactone, and methylpyrazine had sweet, fruity, and cocoa flavors, respectively [47,48,49]. However, the content of these aroma compounds decreased with increasing infection time, resulting in a gradual weakening of sweet potato flavors.

### 3.3. Correlation between VOCs and Physiological Indicators

**Figure 5:**
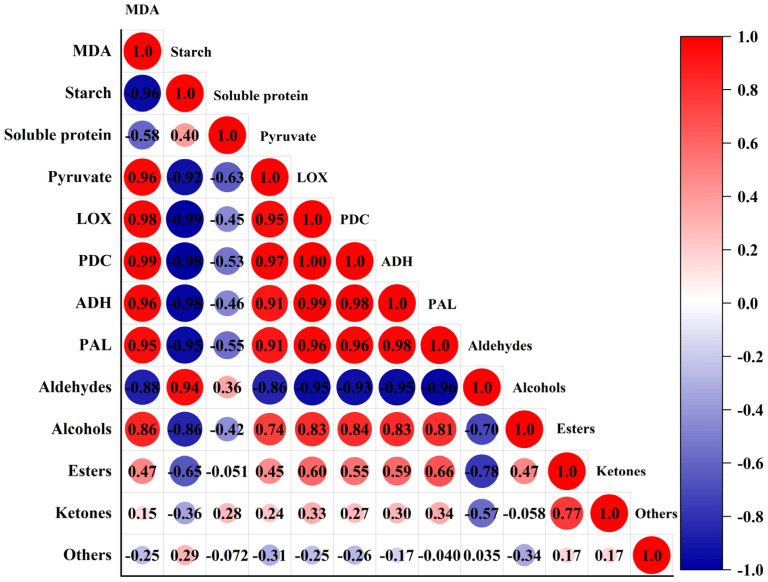
Correlation analysis between volatile organic compounds (VOCs) and physiological indicators of *C. fimbriata*-infected sweet potatoes.

As can be seen from Figure 5, there were positive correlations and negative correlations between VOCs and physiological indicators. A positive correlation meant that the content of VOCs and physiological indicators changed in the same trend, while a negative correlation meant that the content of VOCs and physiological indicators changed in the opposite trend. Aldehydes had the highest positive correlation with starch (r = 0.94, *p* < 0.05) and the highest negative correlation with PAL (r = −0.96, *p* < 0.05). The correlation between aldehydes and MDA, soluble protein, pyruvate, LOX, PDC, and ADH was −0.88, 0.36, −0.86, −0.95, −0.93, and −0.95, respectively. Alcohols had the highest positive correlation with MDA (r = 0.86, *p* < 0.05), and the highest negative correlation with starch (r = −0.86, *p* < 0.05). The correlation with soluble protein, pyruvate, LOX, PDC, ADH, and PAL were −0.42, 0.74, 0.83, 0.84, 0.83, and 0.81, respectively. Esters had the highest positive correlation with PAL (r = 0.66, *p* < 0.05), and the highest negative correlation with starch (r = −0.65, *p* < 0.05). The correlations with MDA, soluble protein, pyruvate, LOX, PDC, and ADH were 0.47, −0.051, 0.45, 0.60, 0.55, and 0.59, respectively. The content of aldehydes and alcohols was greatly affected by the changes in MDA content, starch content, pyruvate content, and activities of LOX, PDC, ADH, and PAL. The ester content was greatly affected by the changes in starch content, LOX activity, and PAL activity. In summary, the changes in VOC content in *C. fimbriata*-infected sweet potatoes were closely related to physiological indicators.

### 3.4. Discrimination of Sweet Potatoes with Different Infection Times Based on PCA

**Figure 6:**
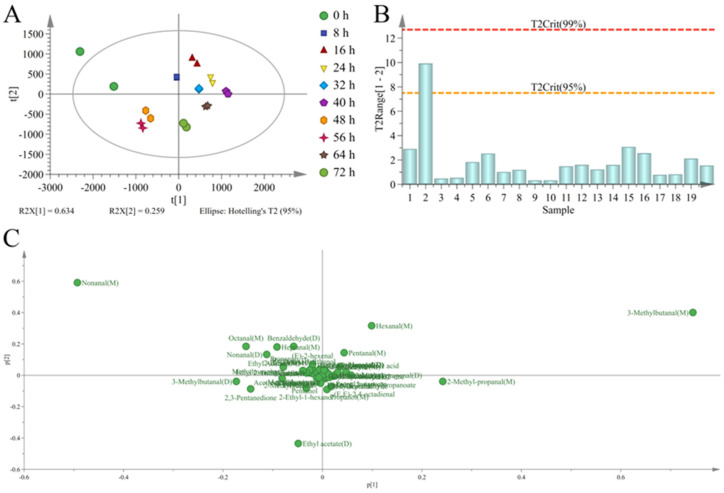
Principal component analysis results of the score chart (**A**), Hotelling’s T^2^ test (**B**), and loading analysis (**C**) of *C. fimbriata*-infected sweet potatoes.

Principal component analysis is an unsupervised dimension reduction analysis method that can reduce the dimensions of original multidimensional data into a small number of comprehensive indicators to reflect the information of original variables and evaluate the regularity and difference between samples [50]. The HS-GC-IMS signal intensity was combined with PCA with R^2^X = 0.893 > 0.5 and Q^2^ = 0.689 > 0.5 values obtained. R^2^X represents the fitting degree of the input variables, and Q^2^ represents the cross-validated prediction ability of the model. The results indicated that the model could explain most of the data, was well-fitted, and there was a good separation between sweet potatoes at 0 h and sweet potatoes from 8 to 72 h (Figure 6A). The red dotted line in Hotelling’s T^2^ test defined a 99% confidence interval that could be used to diagnose outliers (Figure 6B). All samples were within the 99% confidence interval; this indicated good experimental repeatability and a clustering trend [51]. Loading analysis results showed that 3-methylbutanal (M), nonanal (M), 2-methyl-propanal (M), 3-methylbutanal (D), and octanal (M) had a high contribution rate in the PC1 direction (Figure 6C). Meanwhile, 3-methylbutanal (M), nonanal (M), ethyl acetate (D), hexanal (M), and benzaldehyde (D) had a high contribution rate in the PC2 direction. The above compounds had a significant impact on the clustering of sweet potatoes at different infection times. Principal component analysis results showed that sweet potatoes with different infection times had good differentiation; however, there was a large dispersion at 72 h. Therefore, the discriminant analysis model could be optimized to determine differential VOCs.

### 3.5. Discrimination of Sweet Potatoes with Different Infection Times Based on OPLS-DA

**Figure 7:**
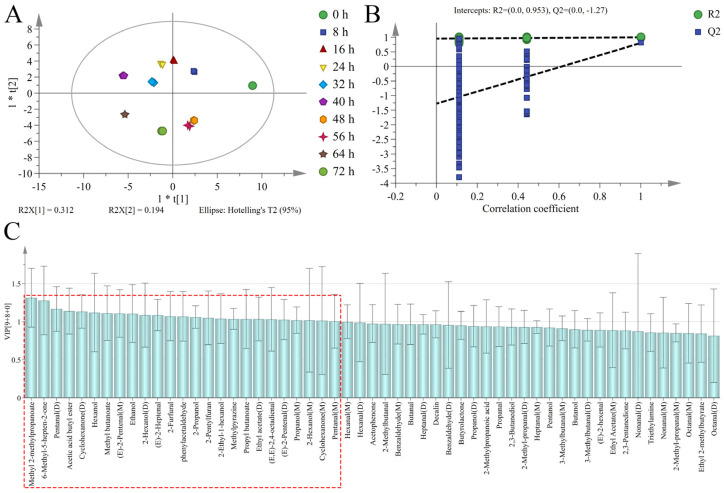
The orthogonal partial least squares discriminant analysis (OPLS-DA) results of the score chart (**A**), permutation test (**B**), and variable influence on projection (VIP) value (**C**) of *C. fimbriata*-infected sweet potatoes.

Orthogonal partial least squares-discriminant analysis can more easily exclude independent variables unrelated to classification and screen out characteristic variables of samples compared with PCA [52]. The HS-GC-IMS signal intensity combined with OPLS-DA resulted in R^2^X = 0.997; R^2^Y = 0.998; and Q^2^ = 0.763 where R^2^X and R^2^Y represent the fitting degree of input variables and class response respectively; while Q^2^ represents the cross-validated prediction ability of the model (Figure 7A). This indicated that the model had good predictive ability. The discrepancy between R^2^Y and Q^2^ is below 0.3 when R^2^X; R^2^Y; and Q^2^ exceeds 0.5. This indicated that the model was well-fitted and the prediction was robust [53]. The separation between sweet potatoes at 0 h and 8 to 72 h indicated that the VOCs of fresh sweet potatoes were significantly different from diseased sweet potatoes. At the same time; the differentiation of diseased sweet potatoes from 8 to 72 h was good. This indicated that the VOCs significantly changed in sweet potatoes with increasing infection time. The permutation test was carried out to verify the reliability of the model to avoid overfitting. Part of the sample class Y was randomly selected 200 times for the permutation test (Figure 7B). The intersection of R^2^ and the vertical axis was (0; 0.953); the intersection of Q^2^ and the vertical axis was (0; −1.27). The intercept value of Q^2^ was −1.27 (Q^2^ < 0)*,* and all the R^2^ and Q^2^ values were projected to the left and below the original values. These results indicated that the model had good stability and prediction ability without overfitting [54]. 

The contribution rate of each variable to the classification could be quantified according to the variable influence on projection (VIP) value based on the constructed OPLS-DA model (Figure 7C). A larger VIP value indicates that there is a greater difference in this variable amongst different groups and the more critical it is for classification. It is generally believed that variables with a VIP value above 1 have an important influence on the classification of the model [55]. There is a total of 25 VOCs with VIP values greater than 1 (as shown in the red box) (Figure 7C). This includes monomers and dimers of some compounds, including methyl 2-methylpropanoate, 6-methyl-5-hepten-2-one, pentanal (D), acetic acid butyl ester, cyclohexanone (D), hexanol, methyl butanoate, (E)-2-pentenal (M), ethanol, 2-hexanol (D), (E)-2-heptenal, 2-furfural, phenylacetaldehyde, 2-propanol, 2-pentylfuran, 2-ethyl-1-hexanol, methylpyrazine, propyl butanoate, ethyl acetate (D), (E,E)-2,4-octadienal, (E)-2-pentenal (D), propanol (M), 2-hexanol (M), cyclohexanone (M), and pentanal (M). The content of these compounds was significantly different in the infection process of sweet potatoes by *C. fimbriata*. These are characteristic compounds that may be used to detect the early stage of infection.

## 4. Conclusions

This study used HS-GC-IMS to analyze the VOCs of *C. fimbriata*-infected sweet potatoes in the early stage. A total of 55 VOCs were identified, including 26 aldehydes, 11 alcohols, 8 esters, 5 ketones, and 5 others. The content of aldehydes and ketones showed a decreasing trend, while the content of alcohols and esters showed an increasing trend. The content of MDA and pyruvate significantly increased with a longer infection time, while the starch content significantly decreased. The soluble protein content initially increased, then decreased, and the activities of LOX, PDC, ADH, and PAL significantly increased. The aldehyde and alcohol content was greatly affected by changes in the MDA content, starch content, pyruvate content, and activities of LOX, PDC, ADH, and PAL. The ester content was greatly affected by the changes in starch content, LOX activity, and PAL activity. Sweet potatoes with different infection times showed good discrimination effects by PCA and OPLS-DA, and 25 differential VOCs were selected as characteristic compounds of *C. fimbriata*-infected sweet potatoes in the early stage. Sweet potatoes infected by *C. fimbriata* are inedible for humans and livestock. The study provided a new detection method for early disease monitoring and warning in sweet potatoes, and laid a theoretical basis for further research on the metabolic mechanism of VOCs.

## Figures and Tables

**Table 1:** Changes of VOC content in *C. fimbriata*-infected sweet potatoes from 0 to 72 h.

No.	Compounds	CAS	Signal Intensity (mV)									
			0 h	8 h	16 h	24 h	32 h	40 h	48 h	56 h	64 h	72 h
	Aldehydes (26)											
1	Propanal	C123386	3835.6 ± 166.6 ^a^	3812.1 ± 38.7 ^ab^	3806.3 ± 0.9 ^ab^	3737.3 ± 15.5 ^ab^	3657.1 ± 25.8 ^bc^	3531.4 ± 18.6 ^cd^	3760.4 ± 48.2 ^ab^	3751.9 ± 5.4 ^ab^	3451.4 ± 15.4 ^d^	3696.7 ± 89.5 ^ab^
2	2-Methyl-propanal (M)	C78842	1327.8 ± 26.9 ^h^	1654.8 ± 15.8 ^f^	1727.6 ± 19.4 ^e^	1895.5 ± 29.8 ^c^	1839.6 ± 17.6 ^d^	2020.1 ± 16.8 ^b^	1510.1 ± 2.9 ^g^	1516.6 ± 33.1 ^g^	2085.9 ± 8.1 ^a^	1839.2 ± 25.0 ^d^
3	2-Methyl-propanal (D)	C78842	62.5 ± 7.7 ^f^	114.8 ± 9.9 ^d^	157.1 ± 6.4 ^c^	188.1 ± 1.4 ^b^	155.9 ± 0.1 ^c^	261.5 ± 6.0 ^a^	102.7 ± 13.5 ^de^	90.7 ± 0.7 ^e^	192.4 ± 7.6 ^b^	163.6 ± 0.2 ^c^
4	Butanal	C123728	161.4 ± 12.1 ^e^	190.5 ± 2.0 ^d^	210.2 ± 6.7 ^bc^	214.0 ± 0.2 ^ab^	207.5 ± 2.5 ^bc^	225.6 ± 3.2 ^a^	196.7 ± 10.7 ^cd^	198.2 ± 2.9 ^cd^	162.7 ± 0.8 ^e^	171.1 ± 1.2 ^e^
5	3-Methylbutanal (M)	C590863	2529.7 ± 106.5 ^h^	3876.5 ± 5.7 ^d^	4355.2 ± 2.2 ^b^	4539.5 ± 22.8 ^a^	4038.0 ± 21.2 ^c^	4540.5 ± 10.5 ^a^	3071.0 ± 12.6 ^f^	2716.3 ± 16.5 ^g^	4050.5 ± 24.1 ^c^	3616.3 ± 40.1 ^e^
6	3-Methylbutanal (D)	C590863	1320.6 ± 53.8 ^a^	976.9 ± 36.6 ^c^	874.5 ± 3.0 ^d^	805.7 ± 14.2 ^e^	991.5 ± 8.3 ^c^	861.3 ± 1.5 ^de^	1180.6 ± 29.8 ^b^	1161.7 ± 17.1 ^b^	878.8 ± 2.2 ^d^	967.1 ± 35.0 ^c^
7	2-Methylbutanal	C96173	255.4 ± 5.2 ^ab^	234.5 ± 6.4 ^bc^	226.6 ± 14.0 ^bc^	208.2 ± 6.5 ^c^	238.1 ± 0.1 ^abc^	234.2 ± 2.4 ^bc^	254.3 ± 24.7 ^ab^	266.8 ± 25.9 ^a^	255.5 ± 4.9 ^ab^	240.1 ± 4.8 ^ab^
8	Pentanal (M)	C110623	2741.6 ± 17.8 ^c^	2877.0 ± 69.0 ^a^	2956.1 ± 35.6 ^a^	2730.3 ± 5.1 ^c^	2766.8 ± 36.9 ^bc^	2866.0 ± 7.7 ^ab^	2582.5 ± 61.2 ^e^	2700.9 ± 41.4 ^cd^	2726.4 ± 10.7 ^c^	2599.3 ± 94.8 ^de^
9	Pentanal (D)	C110623	452.8 ± 24.7 ^e^	483.7 ± 6.3 ^d^	509.0 ± 8.3 ^c^	511.1 ± 2.7 ^c^	542.1 ± 2.4 ^a^	533.6 ± 1.3 ^ab^	470.9 ± 12.9 ^de^	484.0 ± 3.1 ^d^	481.2 ± 1.0 ^d^	512.5 ± 2.3 ^bc^
10	(E)-2-Pentenal (M)	C1576870	86.6 ± 0.4 ^bc^	76.0 ± 3.6 ^c^	76.6 ± 3.6 ^c^	86.6 ± 5.4 ^bc^	100.1 ± 2.8 ^ab^	89.4 ± 3.4 ^bc^	89.2 ± 12.3 ^bc^	81.5 ± 13.3 ^c^	106.0 ± 5.8 ^a^	83.0 ± 2.9 ^c^
11	(E)-2-Pentenal (D)	C1576870	69.2 ± 13.1 ^de^	73.7 ± 0.6 ^cd^	84.7 ± 5.6 ^bc^	128.6 ± 1.9 ^a^	93.3 ± 0.2 ^b^	70.4 ± 0.2 ^de^	73.8 ± 2.2 ^cd^	62.6 ± 11.0 ^de^	68.3 ± 0.4 ^de^	57.0 ± 2.6 ^e^
12	Hexanal (M)	C66251	3987.7 ± 205.8 ^cd^	4227.9 ± 1.3 ^ab^	4231.8 ± 101.6 ^ab^	4061.1 ± 33.1 ^bc^	4376.5 ± 70.9 ^a^	4331.9 ± 29.5 ^a^	3830.0 ± 48.6 ^de^	3809.2 ± 93.4 ^de^	3846.9 ± 2.5 ^cde^	3639.8 ± 124.9 ^e^
13	Hexanal (D)	C66251	2382.2 ± 3.4 ^b^	2394.0 ± 22.5 ^b^	2465.3 ± 38.9 ^b^	2386.3 ± 69.5 ^b^	2441.2 ± 26.5 ^b^	2610.7 ± 21.8 ^a^	2390.7 ± 44.6 ^b^	2377.1 ± 3.4 ^b^	2454.9 ± 4.7 ^b^	2381.9 ± 138.1 ^b^
14	2-Furfural	C98011	376.5 ± 12.1 ^b^	307.8 ± 18.7 ^cd^	288.7 ± 2.9 ^d^	433.5 ± 12.2 ^a^	274.2 ± 24.5 ^de^	246.2 ± 1.7 ^ef^	326.6 ± 0.6 ^c^	330.2 ± 28.1 ^c^	217.5 ± 14.3 ^f^	326.6 ± 13.0 ^c^
15	(E)-2-Hexenal	C6728263	733.0 ± 50.5 ^a^	728.2 ± 11.7 ^ab^	721.6 ± 23.5 ^ab^	668.0 ± 5.9 ^cd^	678.1 ± 22.2 ^bc^	641.8 ± 10.0 ^cde^	644.9 ± 21.2 ^cde^	647.5 ± 15.0 ^cde^	613.8 ± 4.7 ^de^	599.2 ± 23.7 ^e^
16	Heptanal (M)	C111717	1461.3 ± 147.3 ^a^	1351.1 ± 16.2 ^ab^	1251.2 ± 68.7 ^bcd^	1229.5 ± 14.6 ^bcd^	1218.2 ± 18.3 ^bcd^	1158.5 ± 6.2 ^cd^	1266.8 ± 48.0 ^bc^	1130.2 ± 12.7 ^d^	992.3 ± 7.1 ^e^	997.2 ± 30.7 ^e^
17	Heptanal (D)	C111717	265.9 ± 45.1 ^a^	242.4 ± 4.8 ^ab^	214.5 ± 23.4 ^bc^	200.7 ± 7.5 ^bc^	203.0 ± 15.2 ^bc^	191.9 ± 7.2 ^cd^	211.4 ± 14.4 ^bc^	170.9 ± 1.0 ^cd^	156.3 ± 9.2 ^d^	152.9 ± 12.2 ^d^
18	(E)-2-Heptenal	C18829555	232.9 ± 5.7 ^ef^	210.4 ± 7.7 ^f^	263.1 ± 27.6 ^cd^	259.4 ± 1.9 ^cd^	316.1 ± 7.0 ^a^	267.0 ± 2.6 ^c^	304.7 ± 4.6 ^a^	295.9 ± 13.2 ^ab^	276.8 ± 9.0 ^bc^	239.8 ± 2.3 ^de^
19	Benzaldehyde (M)	C100527	325.5 ± 41.6 ^a^	323.9 ± 14.0 ^a^	327.9 ± 3.5 ^a^	339.8 ± 3.1 ^a^	332.9 ± 7.9 ^a^	317.1 ± 3.0 ^a^	316.6 ± 15.4 ^a^	322.1 ± 18.5 ^a^	341.1 ± 1.6 ^a^	320.5 ± 1.5 ^a^
20	Benzaldehyde (D)	C100527	1195.7 ± 208.4 ^a^	967.5 ± 12.9 ^bc^	969.0 ± 54.5 ^bc^	996.3 ± 24.3 ^b^	903.1 ± 1.6 ^bc^	938.4 ± 32.1 ^bc^	843.9 ± 35.0 ^bc^	803.9 ± 23.6 ^c^	801.8 ± 4.9 ^c^	804.2 ± 39.9 ^c^
21	Octanal (M)	C124130	1087.2 ± 209.0 ^a^	750.5 ± 5.7 ^b^	752.2 ± 44.7 ^b^	673.2 ± 14.2 ^bc^	624.0 ± 29.1 ^bcd^	498.1 ± 26.7 ^d^	701.7 ± 28.6 ^bc^	643.8 ± 24.6 ^bcd^	490.4 ± 10.4 ^d^	534.7 ± 9.1 ^cd^
22	Octanal (D)	C124130	139.0 ± 38.3 ^a^	100.5 ± 12.9 ^b^	93.1 ± 1.3 ^b^	79.4 ± 1.8 ^b^	76.0 ± 3.3 ^b^	64.0 ± 4.2 ^b^	97.3 ± 11.5 ^b^	92.1 ± 7.8 ^b^	76.0 ± 13.4 ^b^	91.8 ± 11.9 ^b^
23	Phenylacetaldehyde	C122781	507.4 ± 16.0 ^de^	495.3 ± 16.3 ^e^	535.5 ± 16.2 ^cde^	546.7 ± 6.3 ^bcd^	562.4 ± 7.9 ^abc^	581.5 ± 24.6 ^ab^	584.5 ± 16.4 ^ab^	590.7 ± 26.6 ^a^	526.7 ± 1.5 ^cde^	497.7 ± 17.2 ^e^
24	Nonanal (M)	C124196	3416.4 ± 805.3 ^a^	2360.4 ± 29.6 ^b^	2437.9 ± 121.9 ^b^	2011.8 ± 107.7 ^bcd^	1831.6 ± 8.9 ^bcd^	1530.5 ± 55.2 ^d^	2196.4 ± 135.3 ^bc^	2019.8 ± 59.5 ^bcd^	1693.9 ± 28.3 ^cd^	1659.5 ± 70.5 ^cd^
25	Nonanal (D)	C124196	605.6 ± 293.1 ^a^	316.4 ± 41.7 ^b^	302.6 ± 36.9 ^b^	254.6 ± 16.9 ^b^	239.9 ± 19.5 ^b^	194.1 ± 22.7 ^b^	279.3 ± 15.5 ^b^	250.4 ± 19.1 ^b^	214.2 ± 8.2 ^b^	203.8 ± 8.7 ^b^
26	(E,E)-2,4-Octadienal	C30361285	173.1 ± 57.8 ^e^	205.6 ± 30.0 ^e^	282.6 ± 55.0 ^d^	323.7 ± 9.5 ^bcd^	361.6 ± 8.2 ^abc^	278.0 ± 3.3 ^d^	412.3 ± 9.9 ^a^	375.4 ± 2.9 ^ab^	355.5 ± 12.2 ^abc^	300.3 ± 11.6 ^cd^
	Alcohols (11)											
27	Ethanol	C64175	1104.3 ± 164.5 ^ab^	1061.1 ± 9.0 ^b^	972.9 ± 20.6 ^b^	978.0 ± 19.3 ^b^	1015.2 ± 34.9 ^b^	1116.3 ± 23.7 ^ab^	989.2 ± 46.5 ^b^	972.1 ± 40.7 ^b^	1218.7 ± 3.6 ^a^	980.6 ± 86.3 ^b^
28	2-Propanol	C67630	100.3 ± 14.7 ^bcd^	86.7 ± 0.9 ^de^	93.7 ± 1.0 ^bcd^	88.9 ± 1.4 ^cde^	91.4 ± 2.4 ^cde^	108.3 ± 4.6 ^b^	127.0 ± 3.0 ^a^	121.4 ± 10.3 ^a^	76.9 ± 3.6 ^e^	102.9 ± 4.3 ^bc^
29	Propanol (M)	C71238	1573.1 ± 7.9 ^cd^	1563.6 ± 65.3 ^cd^	1492.6 ± 8.4 ^d^	1627.2 ± 0.9 ^bc^	1520.5 ± 6.3 ^cd^	1555.9 ± 35.3 ^cd^	1623.3 ± 87.2 ^bc^	1559.0 ± 64.0 ^cd^	1709.3 ± 21.7 ^ab^	1793.9 ± 54.5 ^a^
30	Propanol (D)	C71238	202.1 ± 26.0 ^e^	250.4 ± 3.3 ^bc^	273.6 ± 16.7 ^b^	305.4 ± 2.7 ^a^	255.9 ± 1.2 ^bc^	307.7 ± 9.6 ^a^	222.6 ± 10.7 ^de^	217.6 ± 11.8 ^de^	238.5 ± 3.0 ^cd^	253.1 ± 3.9 ^bc^
31	Butanol	C71363	498.8 ± 24.1 ^de^	545.1 ± 5.8 ^bc^	543.3 ± 15.1 ^bc^	526.1 ± 5.0 ^cd^	548.3 ± 1.9 ^bc^	563.4 ± 7.3 ^ab^	488.1 ± 13.2 ^e^	502.4 ± 13.5 ^de^	584.5 ± 10.4 ^a^	527.6 ± 20.9 ^cd^
32	Pentanol	C71410	205.1 ± 12.9 ^de^	205.9 ± 9.7 ^de^	188.2 ± 0.4 ^e^	193.2 ± 1.7 ^e^	233.4 ± 2.1 ^bc^	218.6 ± 6.2 ^cd^	250.1 ± 10.1 ^b^	269.2 ± 2.3 ^a^	249.8 ± 8.5 ^b^	277.4 ± 11.9 ^a^
33	2,3-Butanediol	C513859	319.5 ± 15.5 ^ab^	330.8 ± 1.5 ^a^	336.1 ± 26.4 ^a^	327.9 ± 1.9 ^a^	292.9 ± 3.7 ^bc^	288.5 ± 3.1 ^bc^	287.9 ± 16.3 ^bc^	294.8 ± 22.8 ^bc^	261.4 ± 3.1 ^c^	265.0 ± 12.5 ^c^
34	2-Hexanol (M)	C626937	283.2 ± 95.1 ^a^	205.0 ± 36.6 ^ab^	233.3 ± 76.1 ^ab^	176.1 ± 18.8 ^ab^	174.7 ± 1.2 ^ab^	235.8 ± 12.0 ^ab^	207.8 ± 29.1 ^ab^	220.2 ± 14.4 ^ab^	186.9 ± 37.0 ^ab^	172.6 ± 10.0 ^b^
35	2-Hexanol (D)	C626937	58.8 ± 3.8 ^ab^	56.8 ± 3.9 ^abc^	48.7 ± 4.6 ^cd^	52.9 ± 2.5 ^bc^	57.1 ± 2.8 ^abc^	49.8 ± 0.8 ^c^	61.3 ± 5.9 ^a^	56.3 ± 2.0 ^abc^	41.2 ± 2.3 ^d^	53.1 ± 2.2 ^abc^
36	Hexanol	C111273	117.6 ± 10.1 ^a^	151.3 ± 2.4 ^a^	120.8 ± 5.4 ^a^	123.6 ± 7.9 ^a^	119.0 ± 7.7 ^a^	137.9 ± 8.8 ^a^	128.1 ± 12.0 ^a^	144.0 ± 35.3 ^a^	144.3 ± 23.5 ^a^	136.8 ± 40.3 ^a^
37	2-Ethyl-1-hexanol	C104767	429.6 ± 86.4 ^bcd^	337.2 ± 9.1 ^e^	330.5 ± 17.5 ^e^	355.6 ± 5.5 ^e^	379.2 ± 3.0 ^de^	392.9 ± 4.9 ^cde^	454.1 ± 16.3 ^bc^	484.9 ± 7.9 ^ab^	387.5 ± 24.8 ^cde^	545.7 ± 12.8 ^a^
	Esters (8)											
38	Ethyl acetate (M)	C141786	1402.8 ± 61.0 ^a^	1339.7 ± 1.8 ^b^	1296.5 ± 15.8 ^bc^	1251.5 ± 13.6 ^cd^	1242.6 ± 0.2 ^cd^	1249.7 ± 0.2 ^cd^	1280.7 ± 4.0 ^cd^	1266.5 ± 26.1 ^cd^	1230.3 ± 4.3 ^d^	1290.4 ± 14.4 ^bc^
39	Ethyl acetate (D)	C141786	3411.4 ± 66.4 ^e^	3607.1 ± 26.8 ^bc^	3231.7 ± 87.3 ^f^	3560.4 ± 12.1 ^cd^	3485.5 ± 3.8 ^de^	3710.7 ± 13.5 ^b^	4048.2 ± 11.0 ^a^	4066.4 ± 50.1 ^a^	3663.3 ± 45.4 ^bc^	4047.0 ± 48.5 ^a^
40	Methyl 2-methylpropanoate	C547637	384.8 ± 0.4 ^e^	378.9 ± 2.3 ^e^	384.2 ± 3.4 ^e^	431.2 ± 14.7 ^c^	412.3 ± 5.5 ^d^	447.9 ± 1.2 ^ab^	457.6 ± 6.1 ^a^	382.8 ± 5.9 ^e^	436.8 ± 6.9 ^bc^	401.4 ± 5.2 ^d^
41	Methyl butanoate	C623427	43.0 ± 1.3 ^abc^	35.9 ± 0.1 ^cde^	37.1 ± 5.0 ^bcde^	44.1 ± 0.4 ^ab^	41.0 ± 4.9 ^abcd^	47.1 ± 1.2 ^a^	40.0 ± 4.8 ^abcd^	34.3 ± 3.3 ^de^	32.5 ± 0.7 ^e^	34.0 ± 1.9 ^de^
42	Acetic acid butyl ester	C123864	80.2 ± 1.9 ^bc^	87.1 ± 6.0 ^abc^	75.7 ± 5.3 ^bc^	72.1 ± 1.9 ^c^	91.3 ± 0.8 ^ab^	101.3 ± 2.5 ^a^	77.9 ± 6.2 ^bc^	81.2 ± 16.2 ^bc^	75.7 ± 1.3 ^bc^	70.8 ± 9.6 ^c^
43	Ethyl 2-methylbutyrate	C7452791	218.9 ± 40.7 ^a^	169.7 ± 5.9 ^bc^	170.5 ± 13.7 ^bc^	146.8 ± 6.3 ^bcde^	158.7 ± 3.0 ^bcde^	132.6 ± 8.7 ^de^	178.4 ± 2.5 ^b^	167.4 ± 4.6 ^bcd^	131.1 ± 1.8 ^e^	140.3 ± 9.4 ^cde^
44	Propyl butanoate	C105668	450.9 ± 72.8 ^a^	533.2 ± 9.7 ^a^	510.8 ± 41.7 ^a^	511.8 ± 29.9 ^a^	482.1 ± 19.2 ^a^	54.1 ± 16.9 ^a^	498.9 ± 4.9 ^a^	509.4 ± 63.2 ^a^	478.8 ± 25.5 ^a^	478.8 ± 72.3 ^a^
45	Butyrolactone	C96480	233.0 ± 34.9 ^ab^	222.9 ± 0.3 ^abc^	217.5 ± 24.0 ^abc^	253.2 ± 2.8 ^a^	190.5 ± 0.1 ^cd^	188.2 ± 30.6 ^cd^	215.8 ± 0.2 ^abcd^	207.0 ± 2.6 ^bcd^	175.4 ± 1.7 ^d^	204.0 ± 0.1 ^bcd^
	Ketones (5)											
46	2,3-Pentanedione	C600146	1837.3 ± 89.4 ^a^	1587.4 ± 109.7 ^cd^	1499.3 ± 34.2 ^de^	1379.5 ± 8.8 ^e^	1555.1 ± 8.0 ^cd^	1512.7 ± 0.9 ^de^	1764.8 ± 69.9 ^ab^	1789.7 ± 100.2 ^ab^	1526.3 ± 10.7 ^de^	1678.9 ± 53.2 ^bc^
47	Cyclohexanone (M)	C108941	93.4 ± 4.5 ^ab^	90.7 ± 2.0 ^b^	95.0 ± 6.6 ^ab^	97.7 ± 0.5 ^ab^	98.9 ± 7.6 ^ab^	104.2 ± 6.4 ^a^	94.4 ± 2.3 ^ab^	96.8 ± 5.6 ^ab^	99.8 ± 4.0 ^ab^	93.6 ± 1.4 ^ab^
48	Cyclohexanone (D)	C108941	428.0 ± 18.5 ^cd^	459.4 ± 9.7 ^bc^	471.5 ± 6.6 ^b^	513.0 ± 3.2 ^a^	513.2 ± 6.9 ^a^	485.1 ± 17.9 ^ab^	463.5 ± 16.2 ^b^	518.0 ± 18.2 ^a^	418.5 ± 10.6 ^d^	454.5 ± 24.7 ^bc^
49	6-Methyl-5-hepten-2-one	C110930	580.6 ± 18.8 ^d^	603.9 ± 5.9 ^cd^	536.6 ± 21.0 ^e^	652.3 ± 4.1 ^a^	639.9 ± 5.0 ^ab^	608.1 ± 11.5 ^cd^	596.8 ± 3.5 ^cd^	615.2 ± 17.0 ^bc^	541.4 ± 1.5 ^e^	600.9 ± 17.0 ^cd^
50	Acetophenone	C98862	341.5 ± 10.1 ^ab^	333.9 ± 10.6 ^ab^	305.9 ± 9.4 ^cde^	344.0 ± 9.5 ^a^	328.7 ± 7.9 ^abc^	300.1 ± 19.7 ^de^	335.2 ± 11.5 ^ab^	334.4 ± 7.3 ^ab^	284.8 ± 13.7 ^e^	313.4 ± 12.0 ^bcd^
	Others (5)											
51	Triethylamine	C121448	490.5 ± 22.9 ^a^	425.3 ± 15.3 ^b^	416.0 ± 7.8 ^bcd^	387.4 ± 7.4 ^de^	404.9 ± 11.6 ^bcd^	368.1 ± 6.1 ^e^	422.9 ± 1.6 ^bc^	420.6 ± 22.7 ^bc^	392.1 ± 1.9 ^cde^	406.2 ± 4.9 ^bcd^
52	2-Methylpropanoic acid	C79312	171.1 ± 21.9 ^abcd^	166.3 ± 8.3 ^bcd^	181.9 ± 11.3 ^abc^	199.4 ± 11. 6 ^a^	191.7 ± 11.3 ^ab^	183.9 ± 4.2 ^abc^	184.4 ± 11.7 ^abc^	166.5 ± 13.8 ^bcd^	156.7 ± 1.0 ^cd^	146.1 ± 7.7 ^d^
53	Methylpyrazine	C109080	696.5 ± 23.0 ^a^	542.5 ± 46.1 ^c^	470.7 ± 10.6 ^d^	646.6 ± 2.7 ^b^	456.2 ± 22.4 ^d^	437.9 ± 5.8 ^d^	652.2 ± 24.2 ^ab^	644.5 ± 17.3 ^b^	443.8 ± 6.0 ^d^	556.5 ± 2.2 ^c^
54	2-Pentylfuran	C3777693	186.5 ± 24.9 ^bcd^	178.6 ± 3.0 ^cd^	192.2 ± 16.9 ^bc^	194.9 ± 6.4 ^bc^	215.6 ± 4.0 ^ab^	187.4 ± 7.9 ^bcd^	225.6 ± 2.1 ^a^	214.8 ± 6.0 ^ab^	183.8 ± 18.0 ^cd^	161.0 ± 13.1 ^d^
55	Decalin	C91178	271.9 ± 109.0 ^bc^	331.3 ± 6.0 ^b^	319.1 ± 30.4 ^b^	321.1 ± 8.8 ^b^	280.4 ± 12.2 ^bc^	309.8 ± 26.1 ^b^	213.5 ± 17.0 ^c^	189.8 ± 1.3 ^c^	418.7 ± 14.7 ^a^	256.4 ± 26.6 ^bc^

Values with different lowercase letters in the same row are significantly different (*p* < 0.05).

## Data Availability

The data sets generated for this study are available on request from the corresponding author.
